# Efficient Synthesis of Novel Pyridine-Based Derivatives via Suzuki Cross-Coupling Reaction of Commercially Available 5-Bromo-2-methylpyridin-3-amine: Quantum Mechanical Investigations and Biological Activities

**DOI:** 10.3390/molecules22020190

**Published:** 2017-01-27

**Authors:** Gulraiz Ahmad, Nasir Rasool, Hafiz Mansoor Ikram, Samreen Gul Khan, Tariq Mahmood, Khurshid Ayub, Muhammad Zubair, Eman Al-Zahrani, Usman Ali Rana, Muhammad Nadeem Akhtar, Noorjahan Banu Alitheen

**Affiliations:** 1 Department of Chemistry, Government College University Faisalabad, Faisalabad 38000, Pakistan; Gulchemist35@gmail.com (G.A.); chemistue@gmail.com (H.M.I.); samreengul@gcuf.edu.pk (S.G.K.); nr_308@hotmail.com (M.Z.); 2 Department of Chemistry, COMSATS Institute of Information Technology, University Road, Tobe Camp, 22060 Abbottabad, Pakistan; mahmood@ciit.net.pk (T.M.); khurshid@ciit.net.pk (K.A.); 3 Chemistry Department, Faculty of Science, Taif University, 888-Taif, Saudi Arabia; em-s_z@yahoo.com; 4 Sustainable Energy Technologies Center, College of Engineering, P.O. Box 800, King Saud University, Riyadh 11421, Saudi Arabia; ranausmanali4u@gmail.com; 5 Faculty of Industrial Sciences & Technology, University Malaysia Pahang, Lebuhraya Tun, Razak 26300, Kuantan Pahang, Malaysia; nadeemupm@gmail.com; 6 Faculty of Biotechnology and Biomolecular Sciences, University Putra Malaysia, Serdang, Selangor Darul Ehsan 43400, Malaysia

**Keywords:** Suzuki cross-coupling reaction, pyridine derivatives, density functional theory (DFT), anti-thrombolytic, biofilm inhibition, haemolysis

## Abstract

The present study describes palladium-catalyzed one pot Suzuki cross-coupling reaction to synthesize a series of novel pyridine derivatives **2a**–**2i**, **4a**–**4i**. In brief, Suzuki cross-coupling reaction of 5-bromo-2-methylpyridin-3-amine (**1**) directly or via *N*-[5-bromo-2-methylpyridine-3-yl]acetamide (**3**) with several arylboronic acids produced these novel pyridine derivatives in moderate to good yield. Density functional theory (DFT) studies were carried out for the pyridine derivatives **2a**–**2i** and **4a**–**4i** by using B3LYP/6-31G(d,p) basis with the help of GAUSSIAN 09 suite programme. The frontier molecular orbitals analysis, reactivity indices, molecular electrostatic potential and dipole measurements with the help of DFT methods, described the possible reaction pathways and potential candidates as chiral dopants for liquid crystals. The anti-thrombolytic, biofilm inhibition and haemolytic activities of pyridine derivatives were also investigated. In particular, the compound **4b** exhibited the highest percentage lysis value (41.32%) against clot formation in human blood among all newly synthesized compounds. In addition, the compound **4f** was found to be the most potent against *Escherichia coli* with an inhibition value of 91.95%. The rest of the pyridine derivatives displayed moderate biological activities.

## 1. Introduction

It is generally accepted that cross-coupling reactions catalyzed by transition metals offer facile systematic routes for carbon-carbon bond formation when synthesizing a variety of organic compounds of interest [1]. Among the various transition metals, palladium (Pd) exhibits the highest activity for C–C coupling reactions [2]. The Suzuki-Miyaura cross-coupling reaction provides an appropriate and practical approach for the synthesis of biaryl compounds, due to its ability to tolerate a wide range of functional groups and good reaction yields [3]. In the past, pyridine and its derivatives have been used as starting precursors for several useful compounds due to their availability and stability for direct cross-coupling reactions [4,5,6,7,8,9,10,11,12]. With this approach, Liu and co-workers have described the Suzuki cross-coupling reactions between 2-aryl-1,2,3-triazole-*N*-oxides and pyridine-*N*-oxide with good yield of the final products [5]. Pyridine compounds have a number of biological activities, including anti-tumor [13,14], anti-viral [15,16,17], anti-microbial [18,19], anti-diabetic [20] anti-leishmanial [21] and anti-oxidant [22]. Recently, we have reported the efficient synthesis of a series of 5-aryl-2-bromo-3-hexylthiophene-based compounds and a thorough investigation of their biological activities [23]. The biological importance of heterocyclic compounds inspired us to now synthesize some pyridine derivatives and investigate their biological activities. In the present work, we carried out the successful synthesis of a series of novel pyridine derivatives by making use of Suzuki-Miyaura cross-coupling reactions. These novel pyridine derivatives were further explored by the computational studies using DFT methods and their biological activities were also examined in the course of this study.

## 2. Results and Discussion

### 2.1. Synthesis

**Scheme 1:**
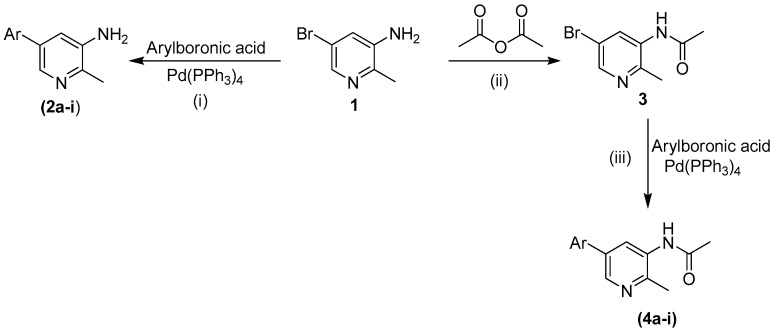
Synthesis of compounds **2a**–**2i**, **3** and **4a**–**4i**; *Reagents and Conditions*: (i) **1** (0.2 g, 1.069 mmol), Pd(PPh_3_)_4_ (5 mol %), arylboronic acid (1.1759 mmol), K_3_PO_4_ (2.318 mmol), 1,4-dioxane:water (2:0.5 mL), 18 h reflux, 90 °C; (ii) **1** (2.0 g, 12.7 mmol), acetic anhydride (1.95 g, 19.1 mmol) in acetonitrile (20 mL); (iii) **3** (0.1 g, 1 mmol), Pd(PPh_3_)_4_ (5 mol %), arylboronic acid (1.1 mmol), K_3_PO_4_ (1.5 mmol), 1,4-dioxane:water (2:0.5 mL), 18 h reflux, 90 °C.

**Figure 1:**
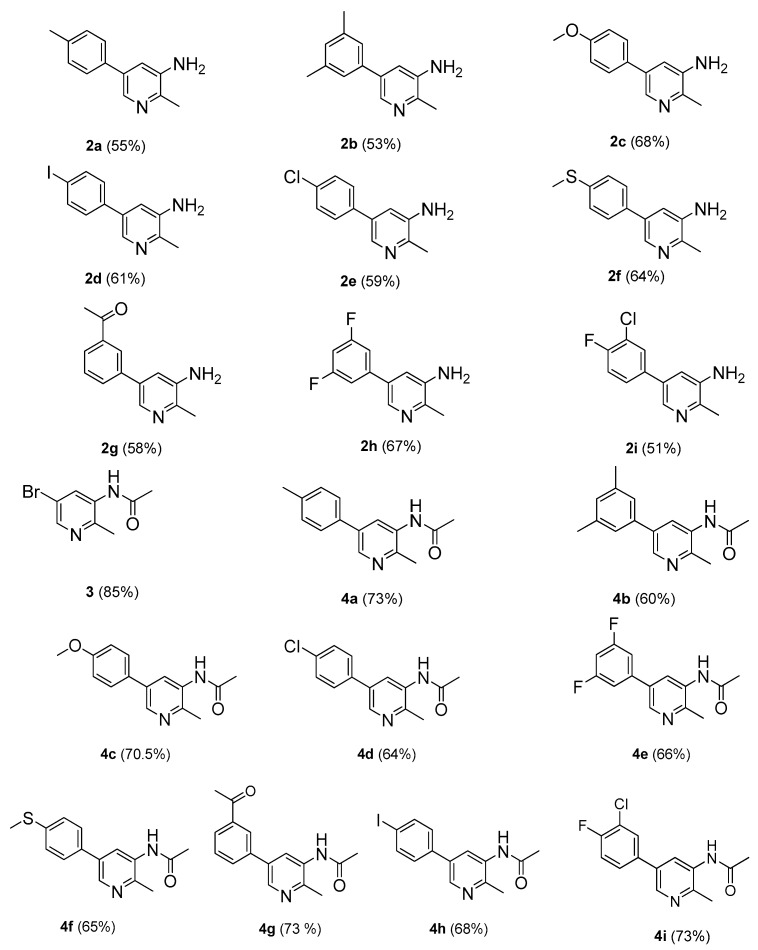
Structures of the newly synthesized pyridine derivatives viz. **2a**–**2i**, **3**, **4a**–**4i**.

Recently, we have reported the successful synthesis of 5-aryl-2-bromo-3-hexylthiophene derivatives by employing the Suzuki cross-coupling reaction [23]. Ikram and co-workers have also reported the application of double Suzuki cross-coupling reactions for the synthesis of 2,5-biaryl-3-hexylthiophene derivatives [24]. In the present work we have utilized the commercially available 5-bromo-2-methylpyridin-3-amine (**1**), which was coupled with a series of arylboronic acids in the presence of tetrakis(triphenylphosphine)palladium(0) as a catalyst and K_3_PO_4_ as a base to produce a series of novel 5-aryl-2-methylpyridin-3-amine molecules **2a**–**2i** (Scheme 1, Figure 1). 

These Suzuki cross-coupling reactions were carried out in a solvent mixture of 1,4-dioxane and water in the volume ratio of 4:1, respectively. As expected, the desired pyridine derivatives were obtained in moderate to good yields. The labile protons in compounds such as carboxylic acids, alcohols and primary amines make them a poor choice for Suzuki coupling reactions [25]. It has been reported that when an amine binds itself with a Pd center, its deprotonation occurs readily with a base such as K_3_PO_4_, which in turn causes a concomitant decrease in the p*K*_a_ [26,27]. Therefore, instead of using carboxylic acids, alcohols and primary amines, we have utilized an amide such as *N*-[5-bromo-2-methylpyridine-3-yl]acetamide (**3**) as the starting material for these Suzuki coupling reactions. This particular amide (*N*-[5-bromo-2-methylpyridine-3-yl]acetamide (**3**)) was synthesized by the reaction of 5-bromo-2-methylpyridin-3-amine (**1**) with acetic-anhydride following Scheme 1 (conditions ii).

The Suzuki cross-coupling reactions of this newly synthesized compound **3** with several arylboronic acids in the presence of Pd(PPh_3_)_4_ and K_3_PO_4_ produced novel pyridine derivatives in moderate to good yields [28]. These cross-coupling reactions were carried out in a solvent mixture of 1,4-dioxane and water (volume ratio ~ 4:1), while the temperature was kept between 85 °C to 95 °C [29]. Interestingly, the *N*-[5-bromo-2-methylpyridine-3-yl]acetamide (**3**) reacted efficiently with various functional group containing arylboronic acids affording the corresponding pyridine derivatives. The effect of different arylboronic acids was studied in the same reaction environment. The experimental results reveal that the electron-donating or electron-withdrawing substituents on the arylboronic acids had no prominent effect on either the reaction rates or the product yields. The structures of all these newly synthesized pyridine based compounds were confirmed by ^1^H-NMR and^13^C-NMR studies.

### 2.2. Density Functional Theory (DFT) Studies

Density functional theory (DFT) studies on all newly synthesized compounds **2a**–**2i** and **4a**–**4i** were carried out to investigate the structure properties relationships in these systems. First of all, the geometry optimization of both series **2a**–**2i** and **4a**–**4i** were simulated using the GAUSSIAN 09 software at B3LYP/6-31G(d,p) level of DFT. Frequency calculations at the same level proved the true optimization, (no imaginary frequency was observed). Structural analysis like frontier molecular orbitals analysis (FMOs), electrostatic potential (ESP) and dipole moment were executed with the help of optimized geometries.

#### 2.2.1. Frontier Molecular Orbital (FMO) Analysis

At present, FMO analysis using DFT methods is becoming vital to explain the reactivity and molecular excitations, as well as the electronic properties of molecular systems [30]. Frontier orbitals (HOMO/LUMO) mainly participate in electronic transitions and their energy gap is used to explain the reactivity and kinetic stability [31]. A low value of HOMO-LUMO energy gap indicates high reactivity and less kinetic stability [32]. The opposite is true if the value of HOMO-LUMO energy gap is high. The FMO analysis of all compounds **2a**–**2i** and **4a**–**4i** was performed at the B3LYP/6-31G(d,p) level of theory, and the respective surfaces are shown in Figure S1 (Supplementary Materials). The corresponding HOMO/LUMO energy values along with energy gap (ΔE) are provided in Table 1. The ΔE values for compounds **2a**–**2i** were found in the range ~4.13–4.86 eV, whereas for the compounds **4a**–**4i**, these values fell in the 4.51–5 eV range. The relatively high values of ΔE for the newly synthesized biphenyl derivatives **4a**–**4i** indicate that these compounds are kinetically more stable than compounds **2a**–**2i**. Among all the newly synthesized derivatives, the compound **4b** showed the value (~5.05 eV), which indicates its higher stability and less reactivity. In contrast, compound **2g** exhibited the lowest ΔE value (~4.13 eV) among all the newly synthesized pyridine derivatives, indicating its poor stability and corresponding high reactivity.

The dispersion of iso-density in the HOMO and LUMO orbitals of compounds **2a**–**2i** displayed similar trends except for **2g**. In the HOMO orbitals, the iso-density dispersion was mainly observed on the biphenyl skeleton, which contained the amino and methyl groups attached to the pyridine ring. In compounds **2a**, **2c**, **2d**, **2e** and **2f**, the groups attached to the *para* position of phenyl ring were involved with the π-electron cloud on the ring, whereas the groups attached to the *meta* position showed no participation with the π-electron cloud, as observed in the case of compounds **2b**, **2g**, **2h** and **2i**. Similarly, in case of the LUMO orbitals, the electronic cloud dispersion in compounds **2a**–**2i** exhibited similar trends except for compound **2g**. The iso-density of the LUMO orbitals reveals that the amino group in the pyridine ring and the groups attached to the *meta* position in the phenyl ring showed no involvement with the π-electron cloud, whereas the groups attached to the *para* position displayed direct participation in the electronic transitions. The dispersion of the iso-density in the HOMO orbitals of compound **2g** was mainly restricted around the pyridine ring, along with the groups attached to it, whereas it was totally shifted to the phenyl ring in the LUMO, indicating a rapid movement of electrons and more reactivity of compound **2g**. The relatively low energy gap between the HOMO and LUMO orbitals of compound **2g** also support this finding.

In the second series of compounds **4a**–**4i**, the iso-density showed a high dependence on the substituents attached to the biphenyl skeleton. In the HOMO orbitals of all compounds **4a**–**4i**, except for **4g**, the iso-density was dispersed around the main skeleton to which substituents were attached at the *para* position. In the HOMO orbitals, a shift in iso-density was observed from the amide group of the pyridine ring toward the pyridine and phenyl ring. Again, the iso-density dispersion in compound **4g** was different. In the HOMO orbitals of compound **4g**, the dispersion was concentrated on the pyridine ring, while in the LUMO, it totally shifted to the phenyl ring. This rapid shifting of electronic cloud indicates the more reactive nature of compound **4g** towards electrophiles when compared to the other compounds of this series **4a**–**4i**.

#### 2.2.2. Reactivity Indices

Reactivity indices like chemical hardness (η), electrophilicity (ω) and electronic chemical potential are excellent tools to describe the chemical hardness, reactivity and stability of compounds [33]. Chemical hardness of any compound can be expressed in terms of the following mathematical equation:

η = (E_HOMO_ − E_LUMO_)/2

Using the equation above the chemical hardness of all derivatives **2a**–**2i** and **4a**–**4i** was calculated and given in Table 2, along with reactivity indices (electrophilicity and electronic chemical potential). From Table 2 it is clear that among all derivatives, **4b** has highest value of η i.e., of 2.52 eV and is the chemically hardest compound among all, whereas **2g** has the lowest value and is chemically soft and more reactive. These findings are consistent with the HOMO-LUMO band gaps of all synthesized derivatives. The electronic chemical potential (µ) value gives an idea about the charge transfer within any compound in its ground state. Mathematically it can be defined by the equation below:

µ = (E_HOMO_ + E_LUMO_)/2

From Table 2, it is clear that **4h** has the highest electronic chemical potential value (4.15 eV), among both series **2a**–**2i** and **4a**–**4i** while **2c** has the lowest chemical potential value. 

The electrophilicity index (ω) is a thermodynamic property that measures the changes in energy when a chemical system becomes saturated by adding electrons. It plays an excellent role in describing the chemical reactivity of a system and is mathematically defined by the following equation:

ω = µ^2^/2η

where µ is the electronic chemical potential, η is chemical hardness. The results from Table 2 indicate that **2b** has the lowest electrophilicity index value and is nucleophilic in nature, whereas **4h** has the highest value i.e., of 3.60 eV and is strongly electrophilic in nature.

#### 2.2.3. Molecular Electrostatic Potential (MEP) Analysis

**Figure 2:**
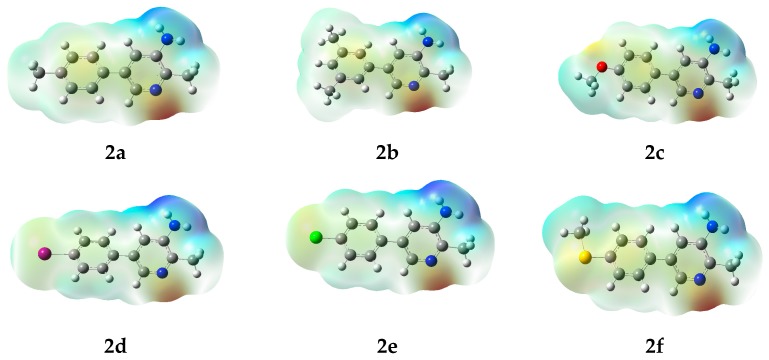

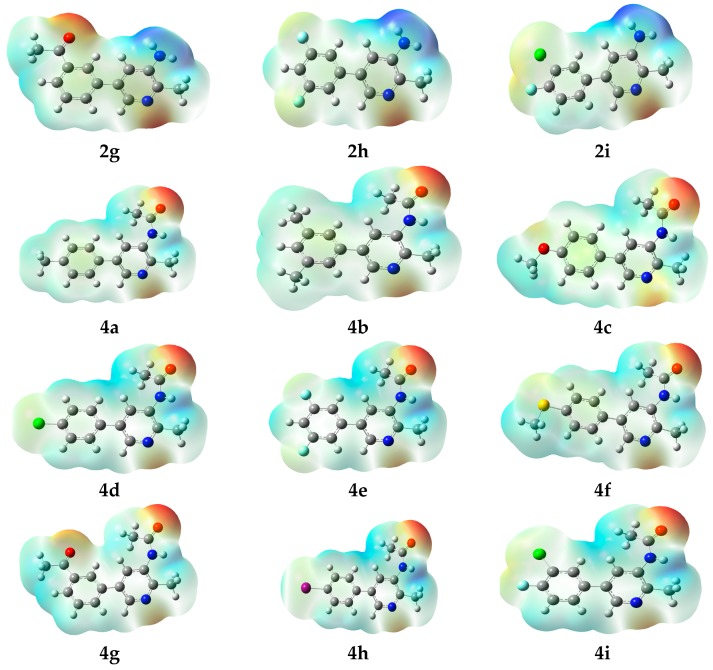
MEP surfaces of the newly synthesized pyridine derivatives viz. **2a**–**2i**, **3**, **4a**–**4i** at B3LYP/6-31G(d,p) level of DFT.

MEP analysis with the help of quantum mechanical methods has been used extensively to explain the reactive sites within a compound [34]. With MEP analysis, the reactive sites can be located by different color codes, such as the red color in a MEP graphic indicates an electron-rich site, blue color indicates an electron-deficient site, while the green color is indicative of a neutral region 32. The MEP analysis of both series of compounds viz. **2a**–**2i** and **4a**–**4i** was carried out using the B3LYP/6-31G(d,p) basis set of DFT. Figure 2 displays the MEP surfaces of the newly synthesized pyridine derivatives viz. **2a**–**2i**, **3**, **4a**–**4i** at B3LYP/6-31G(d,p) level of DFT and graphics, while the corresponding +ve and –ve potential values from the MEP analysis of these compounds are given in Table 3. The MEP surfaces of compounds **2a**–**2i** exhibited similar electronic density trends. In all these compounds, the red color appeared on the pyridine ring nitrogen indicating the electron rich site, while the blue color was concentrated on the amino group, reflecting the electron deficient site.

From the MEP analysis, it is clear that the lone pair of the nitrogen in the pyridine ring was available for electrophiles. In contrast, the lone pair of the amino group exhibited delocalization on the biphenyl skeleton. Similarly, in the case of the second series of compounds **4a**–**4i**, where a hydrogen atom of the amino group was replaced by an acetyl group, the electronic density showed a clear shift toward the acetyl group oxygen, as reflected by red color. The light red color on the nitrogen atom of pyridine still showed its affinity for electrophiles.

#### 2.2.4. Dipole Moment

Biphenyl compounds having restricted rotation around single bonds and a large dipole moment exhibit the potential to influence the liquid crystalline properties, therefore, they are frequently used as chiral dopants for liquid crystals [35]. Biphenyl compounds having greater dipole moments can effectively act as chiral dopants for liquid crystals [36]. The dipole moment of all newly synthesized biphenyl derivatives was calculated at the B3LYP/6-31G(d,p) level of theory and are given in Table 4.

The dipole moment values for compounds **2a**–**2i** were in the ~2.7658–3.9239 Debye range. Compound **2d** displayed the highest dipole moment ~3.7669 Debye, indicating a high suitability of this compound to act as a chiral dopant for liquid crystals. For the second series of compounds **4a**–**4i**, the dipole moment values were recorded in the 1.0557–4.7299 Debye range. Among these compounds, compound **4g** displayed the highest value of dipole moment, indicating its high suitability in terms of chiral dopant properties.

### 2.3. Pharmaceutical Studies

#### 2.3.1. Anti-Thrombolytic Activity

**Figure 3:**
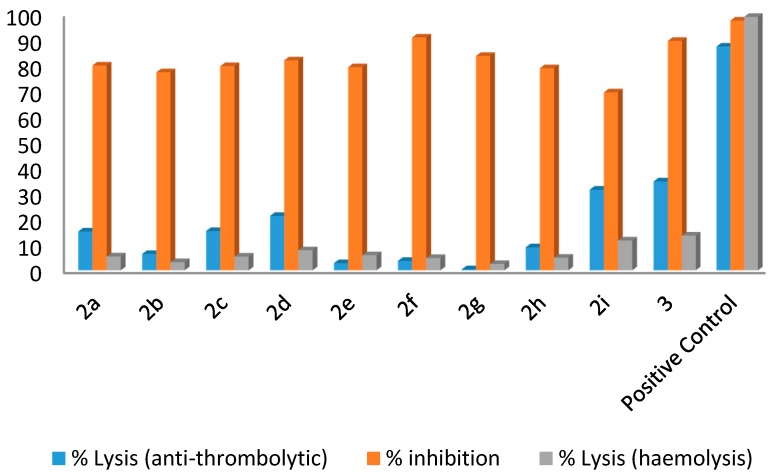
Anti-thrombolytic, biofilm inhibition and haemolytic activities of compounds **2a**–**2i**, **3**.

It has been reported that the thrombotic component is one of the major causes of sudden coronary death and myocardial infarction [37,38]. Hence, we have explored the anti-thrombolytic activity of all newly synthesized compounds. Table 5 displays the anti-thrombolytic activity values for the newly synthesized pyridine derivatives at a concentration of 50 µM/mL. For comparison, streptokinase was used as positive control. The results revealed that compound **2i** exhibited the highest anti-thrombolytic activity (31.61%) among the **2a**–**2i** pyridine derivatives. This high activity indicated that compound **2i** might be related to the presence of two different halogen groups (chloro and flouro) attached at the 3 and 4 positions of the aryl ring, respectively. On the other hand, the compound **2e** showed the lowest activity (2.82%), which could be due to the presence of only one halogen moiety (chloro) at the 4 position of the aryl ring. Furthermore, other derivatives among the **2a**–**2i** series displayed low to moderate anti-thrombolytic activity values, while compound **2g** exhibited no significant activity against clot formation (Figure 3).

**Figure 4:**
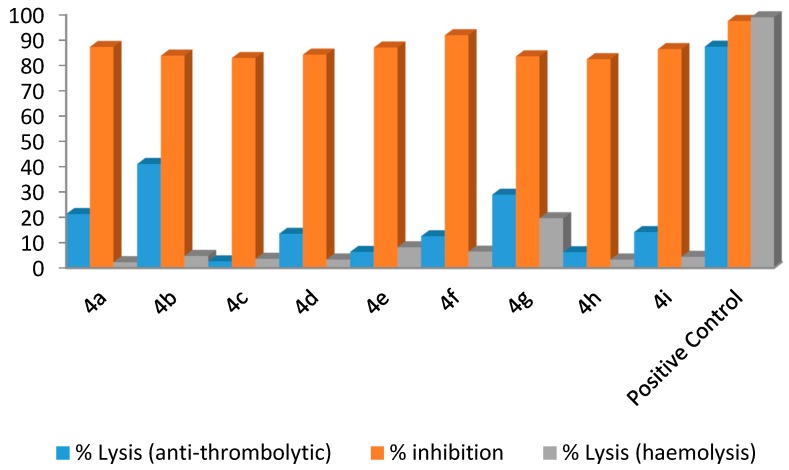
Anti-thrombolytic, biofilm inhibition and haemolytic activities of compounds (**4a**–**4i**).

Among the derivatives **4a**–**4i**, compound **4b** exhibited the highest activity (41.32%) against clot formation in human blood (Table 5, Figure 4). This high activity displayed by **4b** compared to its analogue **2b**, might have its origin in the acyl group attached on the amino group in **4b**. Compound **4c** exhibited the lowest activity against clot formation in human blood among all newly synthesized pyridine derivatives **4a**–**4i**. The results in Table 5 reveal that compounds **4g** and **4a** displayed moderate activity values viz. 29.21% and 21.51%, respectively. Overall, it can be seen that the type of substituents and their position on the aryl group had a significant influence on the anti-thrombolytic activity of the newly synthesized compounds. On the basis of these anti-thrombolytic activity results (Table 5), it is suggested that the pyridine derivatives containing amino groups masked with an acyl group, and substituted aryl rings displayed moderate to good characteristics to inhibit the formation of clots in human blood.

#### 2.3.2. Biofilm Inhibition Activity

Bacterial biofilms are formed when unicellular organisms form a community that adheres itself to a solid surface and is encased in an exopolysaccharide matrix [39]. It is generally accepted that biofilms can limit the effect of medicine to a great extent [40,41,42]. Hence, the anti-biofilm activity of all newly synthesized pyridine-based compounds was evaluated against strains of *Escherichia coli*. Rifampicin was used as standard positive control. The corresponding values of anti-biofilm activity measured for all compounds at a concentration of 50 µM/mL are given in Table 6. In comparison with standard drug rifampicin (97.53%), compound **2f** exhibited the highest activity (90.95 %) among derivatives **2a**–**2i**. This activity displayed by the compound **2f** can be attributed to the existence of the bulky –SMe group attached to the phenyl ring of this compound. Some of the other compounds such as **2g**, **2d**, **2a**, **2c** and **2e** also exhibited good anti-bacterial activities (83.76%, 82.04%, 80.05%, 79.78%, 79.38%, respectively) against *E. coli*. Among all **2a**–**2i** compounds, compound **2i** exhibited the lowest inhibition (69.54%) compared to the positive control rifampicin (Figure 3). 

In the case of pyridine derivatives **4a**–**4i**, the results in Table 6 show that compound **4f** exhibited the highest anti-bacterial activity (91.95%) compared to all other compounds from this series (Figure 4). Since the anti-bacterial activity of these compounds differ with the functional groups, the superior activity exhibited by the compound **4f** could be due to the presence of the thiomethyl group on the phenyl ring in this compound. Several other compounds from this series such as compounds **4a**, **4e**, **4i**, **4d**, **4b**, **4g**, **4c** also exhibited fairly high anti-bacterial activity, evidenced by bio-film inhibition against strains of *E. coli* with values ~87.36%, 87.09%, 86.48%, 84.30%, 83.90%, 83.62 and 82.97%, respectively. The lowest bio-film inhibition value was displayed by compound **4h** and it is plausible that the attached iodo substituent might be the possible cause of this low anti-bacterial activity. Overall, the results for anti-bacterial activity measurements reveal that most of the compounds from both pyridine derivative series **2a**–**2i** and **4a**–**4i** exhibit moderate to good biofilm inhibition values against *E. coli*. In general, it is quite evident that the electron-donating and electron-withdrawing groups attached to different sites of the phenyl ring are responsible for the different antibacterial activity against *E. coli*. The pyridine derivatives **4a**–**4i** exhibited relatively better anti-bacterial activities compared to the **2a**–**2i** series of compounds. Moreover, one approach to enhance the anti-bacterial activity is to mask the amino functional group with an acyl group, as seen earlier in the case of compound **4b**. Hence, pyridine derivatives containing an amino group masked by an acyl group produce potentially useful compounds that can successfully inhibit the formation of biofilms formed by *E. coli*.

#### 2.3.3. Haemolytic Activity

The haemolytic activity of the newly synthesized pyridine derivatives **2a**–**2i** and **4a**–**4i** and compound **3** was also examined. For comparison purposes Triton X-100 was employed as the positive control. The cytotoxicity values for these novel derivatives at a concentration of 50 µM/mL are expressed in Table 7. Among derivatives **2a**–**2i**, compound **2i** exhibited the highest percentage lysis (11.72%) against red blood cells (RBCs). This superior haemolytic activity displayed by compound **2i** might be related with the halogen functional groups (–Cl and –F) attached to the aryl ring in this compound. In contrast, the compound **2g** exhibited the lowest cytotoxicity (2.52%) against RBCs, possibly due to the presence of acyl group attached to the aromatic ring which somehow inhibits the activity of this compound against red blood cells.

In the case of pyridine **4a**–**4i** derivatives, compound **4g** displayed the highest cytotoxicity (19.82%) when compared with the standard drug. Given the presence of two acyl groups in this compound, this remarkably high cytotoxic activity displayed by the compound **4g** was somewhat surprising. From this result, it can be established that upon masking the amino group with an acyl group in the pyridine derivatives, high cytotoxic activity can be achieved against RBCs. The results from Table 7 revealed that the compound **4e** also exhibited good percentage lysis (8.28%) against RBCs. Other pyridine molecules exhibited low to moderate haemolytic activity when compared to Triton X-100. Overall, the results reveal that the haemolytic activity of the newly synthesized pyridine derivatives showed significant variations due to the presence of different substituents at the aromatic ring in these compounds. The high haemolytic activity displayed by most of the newly synthesized pyridine derivatives make them potential active anti-tumor agent candidates [43].

## 3. Experimental Section

### 3.1. General Information

A Buchi B-540 melting point apparatus (Buchi, New Castle, DE, USA) was used to measure the melting points of the newly synthesized pyridine derivatives. For qualitative analysis, an AM-400 NMR spectrometer (Bruker, Billercia, MA, USA) was applied and the ^13^C-NMR and ^1^H-NMR spectra were obtained in CDCl_3_ and CD_3_OD at 400/100 MHz, respectively. All chemicals used in the present work were acquired from Sigma Aldrich (St. Louis, MO, USA) and Alfa Aesar (Ward Hill, MA, USA). EIMS spectra were obtained on a JMS-HX-110 spectrometer (JEOL, Peabody, MA, USA). 

### 3.2. General Procedure for the Synthesis of Compounds ***2a**–**2i***

All compounds were synthesized following the previously reported method [28]. Briefly 5-bromo-2-methylpyridin-3-amine (**1**, 0.2 g with tetrakis(triphenylphosphine)palladium (5 mol %) and 1,4-dioxane (2 mL) were mixed in a Schlenk flask. The reaction mixture was stirred at room temperature for 30 min and then the arylboronic acid (1.1759 mmol), potassium phosphate (2.318 mmol) and water (0.5 mL) were added and the mixture again stirred at 85–95 °C for more than 15 h. After lowering the temperature, the mixture was filtered and diluted with ethyl acetate (50 mL). The excess solvent in the solution was evaporated by rotary evaporator in order to obtain a concentrated solution. Column chromatography was applied to get the desired pure product (silica gel, n-hexane and ethyl acetate). The final products were dried, recrystallized and then characterized using different spectroscopic techniques.

*2-Methyl-5(4-methylphenyl)pyridin-3-amine* (**2a**). m.p.: 208–209 °C. ^1^H-NMR (CDCl_3_) δ 7.99–7.64 (m, 2H-pyridine), 7.41 (m, 2H-Ar), 7.21 (m, 2H-Ar), 2.50 (s, 3H-methyl), 2.41 (s, 3H-methyl); ^13^C-NMR (CDCl_3_ + CD_3_OD) δ 16.7, 21.3, 120.5, 126.8, 128.8, 132.5, 133.8, 134.9, 144.2, 145.8. EI-MS *m/z* (+ion mode): 198, [M − NH_2_]^+^ = 183, [M − NH_2_ and CH_3_]^+^ = 170, [M − NH_2_ and 2CH_3_]^+^ = 155. Anal. calcd for C_13_H_14_N_2_ (198.26): C, 78.74; H, 7.11. Found: C, 78.69; H, 7.02%.

*5-(3,5-Dimethylphenyl)-2-methylpyridin-3-amine* (**2b**). m.p.: 232–233 °C. ^1^H-NMR (CDCl_3_) δ 8.2 (s, 1H-pyridine), 7.6 (m, 2H-Ar), 7.3–7.02 (m, 1H-pyridine, 1H-Ar), 2.51 (s, 3H-methyl), 2.41 (s, 3H-methyl); ^13^C-NMR (CDCl_3_ + CD_3_OD) δ 15.5, 21.5, 120.5, 125.3, 130.6, 132.0, 134.1, 136.4, 138.3, 144.3, 145.8. EI-MS *m/z* (+ion mode): 212, [M − NH_2_]^+^ = 197, [M − NH_2_ and CH_3_]^+^ = 183, [M − NH_2_ and 3CH_3_]^+^ = 140. Anal. calcd for C_14_H_16_N_2_ (212.25): C, 79.19; H, 7.59. Found: C, 78.17; H, 7.56%.

*5-(4-Methoxyphenyl)-2-methylpyridin-3-amine* (**2c**). m.p.: 232 °C. ^1^H-NMR (CDCl_3_) δ 8.12 (s, 1H-pyridine), 7.55 (m, 2H-Ar), 7.31 (m, 1H-pyridine), 7.03 (m, 2H-Ar), 3.9 (s, 3H-methyl), 2.45 (s, 3H-methyl); ^13^C-NMR (CDCl_3_ + CD_3_OD) δ 17.1, 55.6, 114.7, 120.5, 128.4, 129.8, 132.3, 134.3, 144.3, 145.8, 160.3. EI-MS *m/z* (+ion mode): 214, [M − NH_2_]^+^ = 199, [M − NH_2_ and CH_3_]^+^ = 185, [M − OCH_3_]^+^ = 184. Anal. calcd for C_13_H_14_N_2_O (214.21): C, 72.79; H, 6.55. Found: C, 72.75; H, 6.53%.

*5-(4-Iodophenyl)-2-methylpyridin-3-amine* (**2d**). m.p.: 258 °C. ^1^H-NMR (CDCl_3_) δ 8.09 (s, 1H-pyridine), 7.71–7.49 (m, 4H-Ar), 7.15 (s, 1H-pyridine), 2.5 (s, 3H-methyl); ^13^C-NMR (CDCl_3_ + CD_3_OD) δ 15.8, 94.1, 120.1, 130, 132.1, 134.5, 135.7, 138.4, 144.2, 145.5. EI-MS *m/z* (+ion mode): 310, [M − NH_2_]^+^ = 295, [M − NH_2_ and CH_3_]^+^ = 281, [M − I]^+^ = 184. Anal. calcd for C_12_H_11_IN_2_ (310.13): C, 46.47; H, 3.58. Found: C, 46.41; H, 3.52%.

*5-(4-Chlorophenyl)-2-methylpyridin-3-amine* (**2e**). m.p.: 228 °C. ^1^H-NMR (CDCl_3_) δ 8.2 (s, 1H-pyridine), 7.71–7.41 (m, 4H-Ar), 7.11 (s, 1H-pyridine), 2.5 (s, 3H, methyl); ^13^C-NMR (CDCl_3_ + CD_3_OD) δ 15.7, 120.4, 128.3, 129.8, 132, 134.3, 135.3, 144.2, 145.6. EI-MS *m/z* (+ion mode): 310, [M − NH_2_]^+^ = 295, [M − NH_2_ and CH_3_]^+^ = 281, [M − I]^+^ = 184. Anal. calcd for C_12_H_11_ClN_2_ (218.68): C, 66.01; H, 5.07. Found: C, 66.0; H, 5.03%.

*2-Methyl-5-[4-(methylsulfonyl)phenyl]pyridin-3-amine* (**2f**). m.p.: 240 °C. ^1^H-NMR (CDCl_3_) δ 8.23 (s, 1H-pyridine), 7.69–7.11 (m, 4H-Ar, 1H-pyridine), 2.59 (s, 3H, methyl), 2.5 (s, 3H, methyl). ^13^C-NMR (CDCl_3_ + CD_3_OD) δ 14.6, 17, 120.1, 127.2, 128.3, 132.2, 133.4, 135, 139.5, 144, 146.1. EI-MS *m/z* (+ion mode): 230, [M − NH_2_]^+^ = 215, [M − NH_2_ and CH_3_]^+^ = 201, [M − SCH_3_]^+^ = 184. Anal. calcd for C_13_H_14_N_2_S (230.33): C, 66.98; H, 5.99. Found: C, 66.95; H, 5.95%.

*1-[3-(5-Amino-6-methylpyridin-3-yl)phenyl]ethanone* (**2g**). m.p.: 270 °C. ^1^H-NMR (CDCl_3_) δ 8.24 (d, *J* = 3.4 Hz, 1H-Ar), 8.17 (s, 1H-pyridine), 7.96–7.49 (m, 3H-Ar), 7.25 (s, 1H-pyridine), 2.7 (s, 3H-COCH_3_), 2.5 (s, 3H-methyl). ^13^C-NMR (CDCl_3_ + CD_3_OD) δ 16.3, 26.4, 120.5, 126.5, 128.2, 129.7.131.2, 132.8, 134.8, 136.6, 138, 144.5, 146.1, 195. EI-MS *m/z* (+ion mode): 226 [M + H]^+^: [M − NH_2_]^+^ = 211: [M − NH_2_ and CH_3_]^+^ = 197: [M − OCH_3_]^+^ = 184. Anal. calcd for C_14_H_14_N_2_O (226.19): C, 74.27; H, 6.18. Found: C, 74.26; H, 6.17%.

*5-(3,5-Difluorophenyl)-2-methylpyridin-3-amine* (**2h**). m.p.: 208 °C. ^1^H-NMR (CDCl_3_) δ 7.9 (s, 1H-pyridine), 7.57 (m, 1H-pyridine), 7.55–6.82 (m, 3H-Ar), 2.5 (s, 3H, methyl). ^13^C-NMR (CDCl_3_ + CD_3_OD) δ 16.3, 104.8, 111.2, 120, 132.4, 134.5, 139.3, 144.3, 145.8. EI-MS *m/z* (+ion mode): 220 [M + H]^+^: [M − NH_2_]^+^ = 205: [M − NH_2_ and CH_3_]^+^ = 191: [M − 2F]^+^ = 184. Anal. calcd for C_12_H_10_F_2_N_2_ (220.22): C, 65.45; H, 4.56. Found: C, 65.41; H, 4.54%.

*5-(3-Chloro-4-fluorophenyl)-2-methylpyridin-3-amine* (**2i**). m.p.: 240 °C. ^1^H-NMR (CDCl_3_) δ 8.22 (s, 1H-pyridine), 7.72–7.08 (m, 3H-Ar, 1H-pyridine), 2.5 (s, 3H, methyl). ^13^C-NMR (CDCl_3_ + CD_3_OD) δ 16.6, 117.1, 120.4, 121.1, 128, 129.4, 132.2, 133.1, 134.6, 143.9, 145.2. EI-MS *m/z* (+ion mode): 236 [M + H]^+^: [M − NH_2_]^+^ = 221: [M − NH_2_ and CH_3_]^+^ = 207: [M − F]^+^ = 218: [M − Cl]^+^ = 202: [M − F and Cl]^+^ = 184. Anal. calcd for C_12_H_10_ClFN_2_ (236.67): C, 60.90; H, 4.27. Found: C, 60.85; H, 4.22%.

### 3.3. Synthesis of N-[5-Bromo-2-methylpyridine-3-yl]acetamide *(**3**)*

Under a nitrogen atmosphere, a solution of 5-bromo-2-methylpyridine-3-amine (**1**, 2 g), acetic anhydride (1.95 g) and acetonitrile (20 mL) was stirred at 60 °C and treated with 96% H_2_SO_4_ (a few drops). Stirring was continued for another half an hour while the reaction was monitored by TLC. The reaction mixture was evaporated and allowed to cool to room temperature. After cooling, water was added dropwise to form a precipitate. Then the mixture was stirred near one hour around room temperature and then filtered. The impure solid was washed with deionized water and later dried in an oven [44].

*N-[5-Bromo-2-methylpyridine-3-yl]acetamide* (**3**). Yield: 85 %; m.p.: 256 °C. ^1^H-NMR (CDCl_3_) δ 7.8 (s, 1H-pyridine), 7.38 (s, 1H-pyridine), 2.6 (s, 3H, methyl), 2.45 (s, 3H, COCH_3_). ^13^C-NMR (CDCl_3_ + CD_3_OD) δ 16.5, 24.1, 112.3, 127.9, 147.2, 150, 169.1. EI-MS *m/z* (+ion mode): 229 [M + H]^+^: [M − CH_3_]^+^ = 207: [M − Br]^+^ = 150. Anal. calcd for C_8_H_9_BrN_2_O (229.07): C, 41.95; H, 4.01. Found: C, 41.93; H, 3.97%.

### 3.4. General Procedure for the Synthesis of Compounds ***4a**–**4i***

All compounds were synthesized by reported method [28]. *N*-[5-bromo-2-methylpyridine-3-yl]acetamide (**3**, 0.1 g), tetrakis(triphenylphosphine)-palladium (5 mol %) and 1,4-dioxane (2 mL) were placed in the Schlenk flask at room temperature and the mixture was stirred for 30 min . Then the appropriate arylboronic acid (1.1 mmol), potassium phosphate (1.5 mmol) and H_2_O (0.5 mL) were added to the mixture, which was stirred and kept at 85–95 °C for more than 15 h. After reaching room temperature, the mixture was filtered and then diluted with ethyl acetate (50 mL). The excess solvent was evaporated by rotary evaporator in order to obtain a concentrated solution. Column chromatography (silica gel, *n*-hexane and ethyl acetate?) was applied to obtain the desired pure products. The final product was dried and recrystallized and further analyzed using different spectroscopic techniques.

*5-(3-Chloro-4-fluorophenyl)-2-methylpyridin-3-amine* (**4a**). m.p.: 310 °C. ^1^H-NMR (CDCl_3_) δ 8.77 (s, 1H-pyridine), 8.7 (d, *J* =2.5 Hz, 1H-pyridine), 7.6 (m, 2H-Ar), 7.4 (m, 2H-Ar), 2.65 (s, 3H, methyl), 2.5 (s, 3H, methyl), 2.4 (s, 3H, COCH_3_). ^13^C-NMR (CDCl_3_ + CD_3_OD) δ 16.4, 21.5, 24.2, 126.1, 127.6, 129.2, 132.1, 133.2, 134, 136.8, 149.2, 168.2. EI-MS *m/z* (+ion mode): 240 [M + H]^+^: [M − CH_3_]^+^ = 226: [M − 2CH_3_]^+^ = 212. Anal. calcd for C_15_H_16_N_2_O (240.3): C, 74.89; H, 6.69. Found: C, 74.87; H, 6.65%.

*N-[5-(3,5-Dimethylphenyl)-2-methylpyridine-3-yl]acetamide* (**4b**). m.p.: 338 °C. ^1^H-NMR (CDCl_3_) δ 8.6 (s, 1H-pyridine), 8.5 (s, 1H-pyridine), 7.49 (d, 2H-Ar), 7.35 (s, 1H-Ar), 2.49 (s, 3H, methyl), 2.32 (s, 6H, methyl), 2.05 (s, 3H, COCH_3_). ^13^C-NMR (CDCl_3_ + CD_3_OD) δ 16.5, 21.7, 24, 125.2, 126.6, 130.1, 133.5, 136.4, 138.6, 149.3, 167.9. EI-MS *m/z* (+ion mode): 254 [M + H]^+^: [M − CH_3_]^+^ = 240: [M − 3CH_3_]^+^ = 212. Anal. calcd for C_16_H_18_N_2_O (254.33): C, 75.48; H, 7.09. Found: C, 75.45; H, 7.07%.

*N-[5-(4-Methoxyphenyl)-2-methylpyridine-3-yl]acetamide* (**4c**). m.p.: 337 °C. ^1^H-NMR (CDCl_3_) δ 8.27 (s, 1H-pyridine), 7.7 (d, *J* = 2.3 Hz, 1H-pyridine), 7.15–7.05 (m, 4H-Ar), 3.95 (s, 3H, methoxy), 2.6 (s, 3H, methyl), 1.75 (s, 3H, COCH_3_). ^13^C-NMR (CDCl_3_ + CD_3_OD) δ 16.6, 24, 55.6, 114.7, 126.2, 128.3, 129.9, 133.3, 136.8, 149.4, 160.5, 168.3. EI-MS *m/z* (+ion mode): 256 [M + H]^+^: [M − CH_3_]^+^ = 242: [M − 2CH_3_]^+^ = 228. Anal. calcd for C_15_H_16_N_2_O_2_ (256.30): C, 70.29; H, 6.26. Found: C, 70.27; H, 6.24%.

*N-[5-(4-Chlorophenyl)-2-methylpyridine-3-yl]acetamide* (**4d**). m.p.: 316 °C. ^1^H-NMR (CDCl_3_) δ 8.48 (s, 1H-pyridine), 8.22 (s, 1H-pyridine), 7.55 (m, 2H-Ar), 7.45 (m, 2H-Ar), 2.5 (s, 3H, methyl), 2.22 (s, 3H, methyl). ^13^C-NMR (CDCl_3_ + CD_3_OD) δ 16.1, 24.2, 126.6, 128.7, 129.9, 133.4, 134.7, 136.5, 149.2, 169.1. EI-MS *m/z* (+ion mode): 260 [M + H]^+^: [M − CH_3_]^+^ = 247: [M − Cl]^+^ = 226: [M − Cl and CH_3_]^+^ = 212. Anal. calcd for C_14_H_13_ClN_2_O (260.72): C, 64.49; H, 5.08. Found: C, 64.44; H, 5.02%.

*N-[5-(3,5-Difluorophenyl)-2-methylpyridine-3-yl]acetamide* (**4e**). m.p.: 310 °C. ^1^H-NMR (CDCl_3_) δ 8.73 (s, 1H-pyridine), 8.46 (s, 1H-pyridine) 7.29 (m, 2H-Ar), 6.64 (s, 1H-Ar), 2.53 (s, 3H-methyl), 2.04 (s, 3H-methyl). ^13^C-NMR (CDCl_3_ + CD_3_OD) δ 16.6, 24.3, 104.9, 111.2, 126.7, 133.8, 136.5, 139.3, 149.5, 164.8, 168.4. EI-MS *m/z* (+ion mode): 262 [M + H]^+^: [M − CH_3_]^+^ = 248: [M − 2F]^+^ = 226. Anal. calcd for C_14_H_12_F_2_N_2_O (262.23): C, 64.12; H, 4.61. Found: C, 64.08; H, 4.54%.

*N-[2-Methy-5-(4-methylsulfonyl)phenylpyridin-3-yl]acetamide* (**4f**). m.p.: 325 °C. ^1^H-NMR (CDCl_3_) δ 7.69–7.39 (m, 4H-Ar, 2H-pyridine), 2.55 (s, 3H-methyl), 1.7 (s, 3H-methyl). ^13^C-NMR (CDCl_3_ + CD_3_OD) δ 14.7, 16.8, 24.2, 127.1, 128.2, 132.8, 133.8, 136.2, 139.1, 149.6, 168.5. EI-MS *m/z* (+ion mode): 272 [M + H]^+^: [M − CH_3_]^+^ = 258: [M − 2CH_3_]^+^ = 244. Anal. calcd for C_15_H_16_N_2_OS (272.37): C, 66.15; H, 5.92. Found: C, 66.10; H, 5.91%.

*N-[5-(3-Acetylphenyl)-2-methylpyridine-3-yl]acetamide* (**4g**). m.p.: 340 °C. ^1^H-NMR (CDCl_3_) δ 8.33 (s, 1H-pyridine), 8.25 (d, *J* = 1.9 Hz, 1H-pyridine), 8.0 (m, 1H-Ar), 7.80 (m, 1H-Ar), 7.68 (m, 1H-Ar), 7.4 (m, 1H-Ar), 2.71 (s, 3H, methyl), 2.51 (s, 3H, COCH_3_), 2.09 (s, 3H, methyl). ^13^C-NMR (CDCl_3_ + CD_3_OD) δ 16.5, 24.6, 26.3, 125.8, 126.9, 127.7, 128.6, 129.5, 132, 133.7, 136.2, 137.3, 148.6, 167.8, 196.6. EI-MS *m/z* (+ion mode): 268 [M + H]^+^: [M − CH_3_]^+^ = 254: [M − OCH_3_]^+^ = 226. Anal. calcd for C_16_H_16_N_2_O_2_ (268.29): C, 71.59; H, 5.99. Found: C, 71.55; H, 5.96%.

*N-[5-(4-iodophenyl)-2-methylpyridine-3-yl]acetamide* (**4h**). m.p.: 333 °C. ^1^H-NMR (CDCl_3_) δ 8.73 (s, 1H-pyridine), 8.46 (d, *J* = 2.5 Hz, 1H-pyridine), 7.99 (m, 2H-Ar), 7.56 (m, 2H-Ar), 2.53 (s, 3H, methyl), 2.04 (s, 3H, COCH_3_). ^13^C-NMR (CDCl_3_ + CD_3_OD) δ 16.7, 23.9, 94.5, 126.3, 128.2, 130.5, 133.6, 135.7, 136.9, 138.4, 149.1, 168.8. EI-MS *m/z* (+ion mode): 352 [M + H]^+^: [M − CH_3_]^+^ = 338: [M − F]^+^ = 226: [M − F and CH_3_]^+^ = 212. Anal. calcd for C_14_H_13_IN_2_O (352.17): C, 47.75; H, 3.72. Found: C, 47.71; H, 3.67%.

*N-[5-(3-chloro-4-fluorophenyl)-2-methylpyridine-3-yl]acetamide* (**4i**). m.p.: 325 °C. ^1^H-NMR (CDCl_3_) δ 8.2 (s, 1H-pyridine) 7.71 (m, 1H-pyridine), 7.5 (m, 1H-Ar), 7.35 (d, *J* = 8.2 Hz, 1H-Ar), 7.09 (d, *J* = 8.1, 1H-Ar), 2.45 (s, 3H, methyl), 1.7 (s, 3H, COCH_3_). ^13^C-NMR (CDCl_3_ + CD_3_OD) δ 15.9, 24.4, 117.8, 126.3, 127.9, 129.5, 132.9, 135.5, 136.9, 150, 158.1, 167.5. EI-MS *m/z* (+ion mode): 279 [M + H]^+^: [M − CH_3_]^+^ = 265: [M − F]^+^ = 261: [M − Cl]^+^ = 244: [M − Cl and F]^+^ = 226. Anal. calcd for C_14_H_12_ClFN_2_O (278.71): C, 60.33; H, 4.34. Found: C, 60.27; H, 4.29%.

### 3.5. Computational Methods

Density functional theory calculations were executed with the help of the GAUSSIAN 09 software [45] and results/graphics were analyzed with the help of Gauss view 05 [46]. Energy minima geometries of compounds **2a**–**2i** and **4a**–**4i** were optimized without any symmetry constraints at hybrid B3LYP (Becke 3-Parameter, Lee, Yang and Parr) method using 6-31G(d,p) basis set. Frequency analysis was performed at the same level of theory, in order to confirm true optimization. Frontier molecular orbitals (FMOs) analysis, molecular electrostatic potential (MEP) analysis and dipole measurements were executed by using the B3LYP/6-31G(d,p) basis set and at DFT level of theory.

### 3.6. Anti-Thrombolytic Assay

The anti-thrombolytic activity of the novel pyridine derivatives was determined by applying a previously reported method [26]. Human blood was taken from volunteers and kept in sterile microcentrifuge tubes. The clot formation in blood was done, when microcentrifuge tubes having 500 μL blood were incubated for the time of 45 min at optimum temperature (37 °C). After clot formation, the serum was separated and 100 μL test sample solution from the compound (**2a**–**2i**, **3**, **4a**–**4i**) at a concentration of 50 µM/mL were transferred in microcentrifuge tubes. After adding the samples, the tubes were incubated again for the duration of more than 90 min at 37 °C. To act as a positive control, streptokinase was used in this assay, while water was used as a negative control. The final results, shown by test samples against clot formation, were measured in percentage. This study was approved by the Board of studies Department of Chemistry and Director advanced studies, Govt. College University, Faisalabad (DAS600).

### 3.7. Biofilm Inhibition Assay by Microliter-Plate Method

The anti-bacterial activity of new pyridine molecules was determined by applying a reported method [25]. Test sample solutions (100 μL) from the compounds **2a**–**2i**, **3**, **4a**–**4i** at a concentration of 50 µM/mL along with bacterial suspension (20 μL) were placed in sterile 96-well plastic tissue culture plates, which were later incubated for one day at 37 °C. After incubation, phosphate buffer (220 μL) was applied to treat the content of each plate thrice. The plates were shaken vigorously to eliminate non-adherent bacteria and the remaining bacteria were fixed with methanol solution (220 μL, 99%). After drying, the plates were stained by using Crystal Violet solution (220 mL, 50%) for 5 min. Tap water was used to remove the excess stain from plates. Glacial acetic acid (220 μL, 33%) was then applied in order to resolubilise the dye attached to adherent cells. The OD value was determined for each well at 630 nm by a micro-plate reader (BioTek, Winooski, VT, USA).

### 3.8. Haemolytic Assay

The synthesized molecules were evaluated for their haemolytic activity following a previously reported method [47]. In brief, heparinized human blood (3 mL) was taken in sterile Falcon tubes. The tubes were centrifuged at 850 rpm for 5 min. The upper blood layer was taken out and the pellet was washed three times. With the help of ice cold phosphate buffered saline (PBS, 20 mL), the red blood cells were counted via a haemocytometer. The red blood cells were kept on ice and diluted using the pure phosphate buffer saline to 7.068 × 10^8^ cells per mL. Next each compound **2a**–**2i**, **3**, **4a**–**4i** (20 μL) was placed in a 2 mL microfuge tube. Triton X-100 with 100% lysis was used as positive control. For the negative control, phosphate buffered saline with 0.07% lysis was used. The contents of the microfuge tubes were diluted after adding the suspension of blood cells (180 μL). At a temperature of 37 °C, the tubes were incubated for 40 min. After that, the tubes were kept on ice for 5–7 min and then centrifuged at 1315 rpm for 6 min. From this incubated tube, 100 μL of the upper layer was separated, diluted by using ice cold phosphate buffered saline (900 μL). All the test sample solutions (200 μL) were placed into 96-well plates. The absorption of each sample was measured by a µ-Quant instrument at 576 nm. This study was approved by the Board of studies Department of Chemistry and Director advanced studies, Govt. College University, Faisalabad (DAS600).

## 4. Conclusions

The present study describes the efficient synthesis in moderate to good yield of a series of novel pyridine derivatives by the application of palladium catalyzed Suzuki cross-coupling reactions of 5-bromo-2-methylpyridin-3-amine (**1**) and *N*-[5-bromo-2-methylpyridine-3-yl]acetamide (**3**) with several arylboronic acids. Frontier molecular orbitals (FMO) analysis reveals that the spread of isodensity was mainly concentrated on the aromatic and pyridine rings. The HOMO/LUMO band gap studies for all the newly synthesized systems reveal that compound **2g** exhibited the lowest band gap (~4.14 eV), while compound **4b** displayed the highest band gap (~5.05 eV), indicating that compound **2g** is the most reactive and **4b** the least reactive among all the newly synthesized compounds. The chemical hardness of **4b** has the highest value (2.52 eV), whereas **2g** displayed the lowest value, chemically softness and reactivity. Among all derivatives **4h** has the highest value of electronic chemical potential (4.15 eV). The electrophilicity index described the electrophilicity and nucleophilicity quantitatively (**2b** is more nucleophilic and **4b** is more electrophilic). The MEP analysis showed that the negative potential was mainly concentrated on the pyridine ring nitrogen and the positive potential in the compounds **2a**–**i** was mostly concentrated on the amino group. The highest dipole moment value was exhibited by **4g** (4.7299 Debye), indicating its strong potential to act as a chiral dopant for liquid crystals. Further studies on the biological activities of these compounds such as anti-thrombolytic, biofilm inhibition and haemolytic activities revealed the moderate to good biological activities of these newly synthesized pyridine derivatives. Compound **4b** exhibited the highest anti-thrombolytic activity (~41.32%) against clot formation among all the newly synthesized compounds. In addition, compound **4f** exhibited the highest antibacterial activity (~91.95%) against bacterial biofilm formation. In this study, the biological screening results established the effect of different functional groups present at the aromatic ring. In summary, the biological activity studies suggest the potential use of these novel pyridine derivatives as useful compounds for several applications.

## Figures and Tables

**Table 1:** The values of HOMO and LUMO energies for compounds **2a**–**2i** and **4a**–**4i** along with the energy gaps (ΔE). All energy values are reported in eV unit.

Compound	HOMO	LUMO	ΔE
**2a**	−5.51	−0.67	4.84
**2b**	−5.51	−0.65	4.86
**2c**	−5.38	−0.53	4.85
**2d**	−6.03	−1.40	4.63
**2e**	−5.72	−0.98	4.74
**2f**	−5.39	−0.74	4.65
**2g**	−5.66	−1.52	4.14
**2h**	−5.80	−1.09	4.71
**2i**	−5.66	−1.52	4.14
**4a**	−6.08	−1.07	5.01
**4b**	−6.10	−1.05	5.05
**4c**	−5.82	−0.95	4.87
**4d**	−6.30	−1.37	4.93
**4e**	−6.42	−1.47	4.95
**4f**	−5.71	−1.13	4.58
**4g**	−6.28	−1.75	4.53
**4h**	−6.55	−1.76	4.79
**4i**	−6.38	−1.39	4.99

**Table 2:** Reactivity indices of compounds **2a**–**2i** and **4a**–**4i**.

Compound	η (eV)	µ (eV)	ω (eV)
**2a**	2.42	3.09	1.97
**2b**	2.43	3.08	1.95
**2c**	2.42	2.95	1.79
**2d**	2.31	3.71	2.97
**2e**	2.37	3.35	2.36
**2f**	2.32	3.06	2.01
**2g**	2.07	3.59	3.11
**2h**	2.35	3.44	2.51
**2i**	2.07	3.59	3.11
**4a**	2.5	3.57	2.54
**4b**	2.52	3.57	2.52
**4c**	2.43	3.38	2.35
**4d**	2.46	3.83	2.98
**4e**	2.47	3.94	3.14
**4f**	2.29	3.42	2.55
**4g**	2.26	4.01	3.55
**4h**	2.39	4.15	3.6
**4i**	2.49	3.88	3.02

**Table 3:** The +ve and –ve potential values from the MEP analysis of compounds **2a**–**2i** and **4a**–**4i**.

Compound	+ve Potential	−ve Potential
**2a**	−0.05351	0.05351
**2b**	−0.05365	0.05365
**2c**	−0.05348	0.05348
**2d**	−0.05401	0.05401
**2e**	−0.05124	0.05124
**2f**	−0.05285	0.05285
**2g**	−0.05037	0.05037
**2h**	−0.05193	0.05193
**2i**	−0.05198	0.05198
**4a**	−0.05573	0.05573
**4b**	−0.05579	0.05579
**4c**	−0.05643	0.05643
**4d**	−0.05268	0.05268
**4e**	−0.05207	0.05207
**4f**	−0.05527	0.05527
**4g**	−0.05600	0.05600
**4h**	−0.05275	0.05275
**4i**	−0.05230	0.05230

**Table 4:** The values of dipole moment (Debye) for the newly synthesized pyridine derivatives viz. **2a**–**2i**, **3**, **4a**–**4i** calculated at B3LYP/6-31G(d,p) level of DFT.

Compounds	Dipole Moment (Debye)
**2a**	3.0645
**2b**	3.0012
**2c**	2.6799
**2d**	3.7669
**2e**	3.6042
**2f**	3.9239
**2g**	2.7658
**2h**	3.7223
**2i**	3.6598
**4a**	3.3070
**4b**	3.3294
**4c**	4.1817
**4d**	1.0557
**4e**	1.1464
**4f**	3.5552
**4g**	4.7299
**4h**	1.4438
**4i**	2.0915

**Table 5:** Anti-thrombolytic assay of compounds **2a**–**2i**, **3** and **4a**–**4i** at a concentration of 50 µM/mL.

Compounds	% Lysis
**2a**	15.2 ± 0.05
**2b**	6.43 ± 0.06
**2c**	15.4 ± 0.19
**2d**	21.3 ± 0.21
**2e**	2.82 ± 0.33
**2f**	3.72 ± 0.03
**2g**	0.43 ± 0.22
**2h**	9.04 ± 0.31
**2i**	31.6 ± 0.86
**3**	34.8 ± 0.03
**4a**	21.5 ± 0.04
**4b**	41.3 ± 0.43
**4c**	2.69 ± 0.16
**4d**	13.6 ± 0.09
**4e**	6.43 ± 0.21
**4f**	12.6 ± 0.15
**4g**	29.2 ± 0.03
**4h**	6.31 ± 0.41
**4i**	14.3 ± 0.03
Streptokinase	87.45

**Table 6:** Biofilm inhibition assay of the compounds (**2a**–**2i**, **3** and **4a**–**4i**) at a concentration of 50 µM/mL.

Compounds	% Inhibition
**2a**	80.05 ± 0.001
**2b**	77.39 ± 0.040
**2c**	79.78 ± 0.007
**2d**	82.04 ± 0.360
**2e**	79.38 ± 0.030
**2f**	90.95 ± 0.023
**2g**	83.76 ± 0.051
**2h**	78.98 ± 0.212
**2i**	69.54 ± 0.291
**3**	89.63 ± 0.213
**4a**	87.36 ± 0.031
**4b**	83.90 ± 0.112
**4c**	82.97 ± 0.023
**4d**	84.30 ± 0.121
**4e**	87.09 ± 0.321
**4f**	91.95 ± 0.291
**4g**	83.62 ± 0.521
**4h**	82.52 ± 0.02
**4i**	86.48 ± 0.032
Rifampicin	97.53 ± 0.021

**Table 7:** Heamolytic assay of the compounds (**2a**–**2i**, **3** and **4a**–**4i**) at a concentration of 50 µM/mL.

Compounds	% Lysis
**2a**	5.45 ± 0.02
**2b**	3.21 ± 0.51
**2c**	5.40 ± 0.09
**2d**	7.87 ± 0.21
**2e**	5.92 ± 0.05
**2f**	4.82 ± 0.03
**2g**	2.52 ± 0.03
**2h**	4.94 ± 0.11
**2i**	11.7 ± 0.17
**3**	13.6 ± 0.57
**4a**	2.32 ± 0.04
**4b**	4.82 ± 0.04
**4c**	3.67 ± 0.06
**4d**	3.39 ± 0.29
**4e**	8.28± 0.14
**4f**	6.55 ± 0.03
**4g**	19.82 ± 0.37
**4h**	3.39 ± 0.04
**4i**	4.48 ± 0.03
Triton X-100	99

## Supplementary Materials

Supplementary materials can be accessed at: http://www.mdpi.com/1420-3049/22/2/190/s1.
