# Supplementary material for: Efficient Synthesis of Novel Pyridine-Based Derivatives via Suzuki Cross-Coupling Reaction of Commercially Available 5-Bromo-2-methylpyridin-3-amine: Quantum Mechanical Investigations and Biological Activities
Source: Molecules. 2017 Jan 27;22(2):190. doi: 10.3390/molecules22020190 (PMC6155797; doi:10.3390/molecules22020190)
Supplement: Supplementary file 1 [file molecules-22-00190-s001.pdf]

Supplementary Materials: Efficient Synthesis of Novel Pyridine-Based Derivatives via Suzuki Cross-Coupling Reaction of Commercially Available 5-Bromo-2-methylpyridin-3-amine: Quantum Mechanical Investigations and Biological Activities

Gulraiz Ahmad, Nasir Rasool, Hafiz Mansoor Ikram, Samreen Gul Khan, Tariq Mahmood, Khurshid Ayub, Muhammad Zubair, Eman Al-Zahrani, Usman Ali Rana, Muhammad Nadeem Akhtar and Noorjahan Banu Alitheen

S. No.	HOMO	LUMO	S. No.	HOMO	LUMO
2a	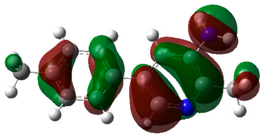	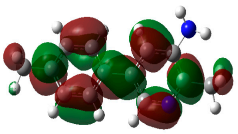	4a	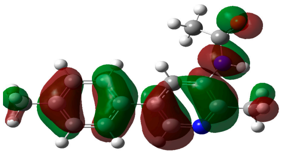	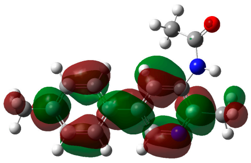
2b	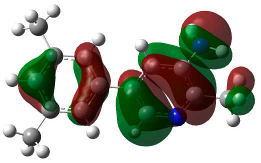	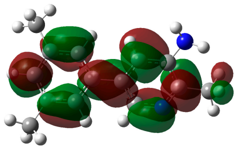	4b	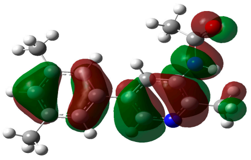	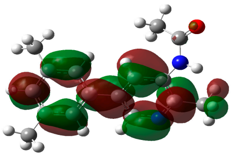
2c	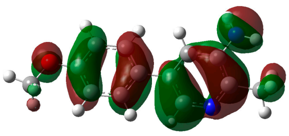	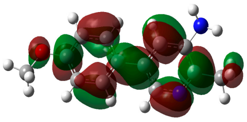	4c	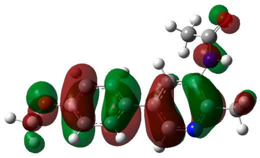	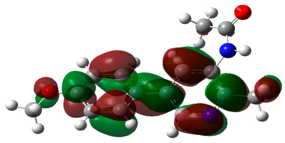
2d	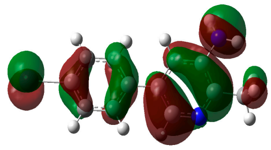	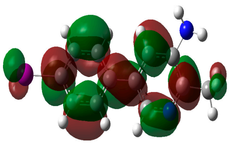	4d	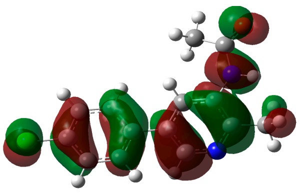	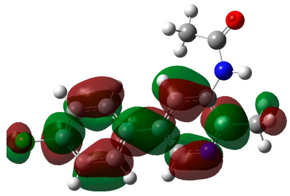

**2e**	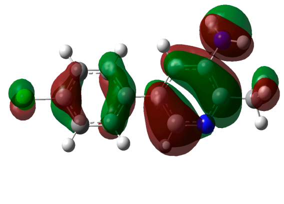	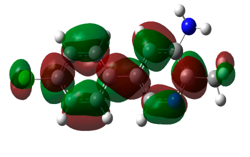	**4e**	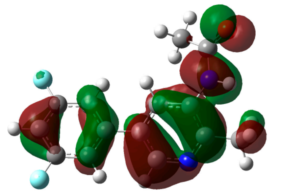	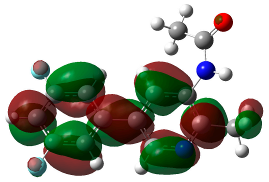
**2f**	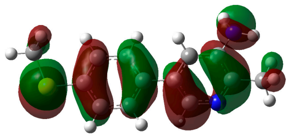	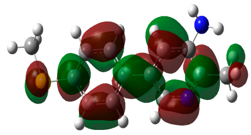	**4f**	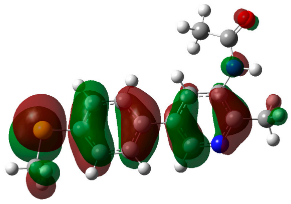	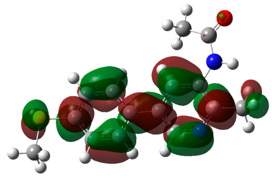
**2g**	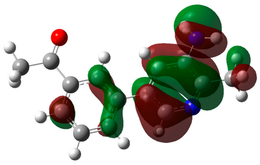	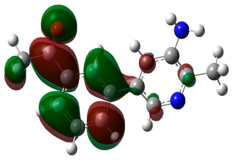	**4g**	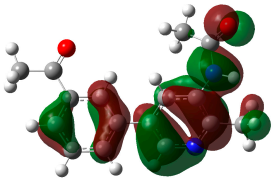	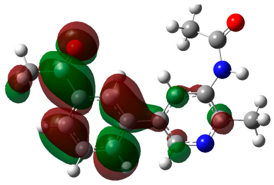
**2h**	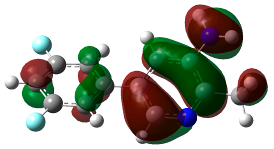	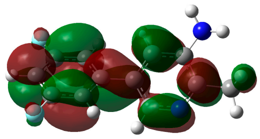	**4h**	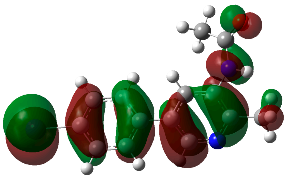	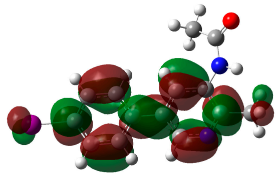
**2i**	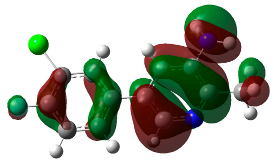	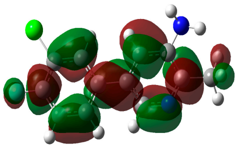	**4i**	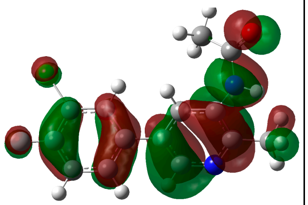	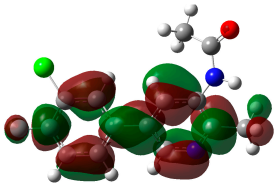

Figure S1. FMOs surfaces of **2a–i** and **4a–i**.
